# HIV-related proteins prolong macrophage survival through induction of Triggering receptor expressed on myeloid cells-1

**DOI:** 10.1038/srep42028

**Published:** 2017-02-09

**Authors:** Zhihong Yuan, Xian Fan, Bashar Staitieh, Chetna Bedi, Paul Spearman, David M Guidot, Ruxana T Sadikot

**Affiliations:** 1 Department of Veterans Affairs, Atlanta VAMC, Decatur, GA 30033, USA; 2 Division of Pulmonary, Allergy, Critical Care & Sleep Medicine, Emory University, Atlanta, GA 30322, USA; 3 Deparment of Pediatrics, Pediatric Infectious Diseases, Emory University, Atlanta, GA 30322, USA

## Abstract

Triggering receptor expressed on myeloid cells-1(TREM-1) is a member of the superimmunoglobulin receptor family. We have previously shown that TREM-1 prolongs survival of macrophages treated with lipoolysaccharide through Egr2-Bcl2 signaling. Recent studies suggest a role for TREM-1 in viral immunity. Human immunodeficiency virus-1 (HIV) targets the monocyte/macrophage lineage at varying stages of infection. Emerging data suggest that macrophages are key reservoirs for latent HIV even in individuals on antiretroviral therapy. Here, we investigated the potential role of TREM-1 in HIV latency in macrophages. Our data show that human macrophages infected with HIV show an increased expression of TREM-1. In parallel, direct exposure to the HIV-related proteins Tat or gp120 induces TREM-1 expression in macrophages and confers anti-apoptotic attributes.NF-κB p65 silencing identified that these proteins induce TREM-1 in p65-dependent manner. TREM-1 silencing in macrophages exposed to HIV-related proteins led to increased caspase 3 activation and reduced Bcl-2 expression, rendering them susceptible to apotosis. These novel data reveal that TREM-1 may play a critical role in establishing HIV reservoir in macrophages by inhibiting apoptosis. Therefore, targeting TREM-1 could be a novel therapeutic approach to enhance clearance of the HIV reservoir, at least within the macrophage pools.

Human immunodeficiency virus (HIV) continues to remain a major global health concern with 34 million individuals living with the infection^1^. Although combination antiretroviral therapy is effective at preventing disease progression, it fails to eradicate virus infection which persists in latent forms^2^,^3^. HIV targets monocyte/macrophage lineage at varying stages of infection and in the chronic phase of infection all of the actors of the immune system may be in a state of activation^4^,^5^,^6^,^7^. We and others have shown that macrophages are key reservoirs for latent HIV even in individuals on antiretroviral therapy^8^,^9^. HIV interacts through multiple signaling pathways to reprogram the transcriptome and the proteome of host cells^10^,^11^,^12^,^13^,^14^,^15^,^16^. It is evident that persistent HIV impairs major functions of macrophages but the molecular basis for this defect remains to be fully characterized^17^,^18^.

TREM proteins (Triggering receptors expressed on myeloid cells) are a family of immunoglobulin cell surface receptors expressed on myeloid cells^19^,^20^. These receptors are capable of regulating various immunological events in both innate and adaptive immune cells^21^. TREM-1 is a transmembrane glycoprotein and bears a single extracellular immunoglobulin domain. Human TREM-1 is a 30 kDa glycoprotein and consists of an extracellular domain, a transmembrane region which contains a conserved lysine residue and a short cytoplasmic domain which lacks any signaling motif. In addition to microbial products which activate TREM-1, recent studies have identified potential ligands for TREM-1 including CD177, PGLYRP1 and HMGB1^21^,^22^,^23^. We and others have also shown that the expression of TREM-1 is modulated by prostaglandins such as PGE_2_ and PGD_2_^24^,^25^. Upon ligation, TREM-1 associates with an aspartate residue in the immunoreceptor tyrosine-based activation motif (ITAM) of the adaptor protein DNAX activation protein 12 (DAP-12). Activation of DAP12-coupled receptors results in transient tyrosine phosphorylation of ITAM chains, acivation of the tyrosine kinase Syk, extracellular signal-regulated kinases 1/2 (ERK1/2), and phosphatidylinositol 3-kinase with an increase in intracellular calcium and pro-inflammatory cytokine secretion through activation of NF-κB^21^,^26^.

The observation that TREM-1 is an amplifier of TLR and NLR induced inflammation led to studies of TREM-1 in animal models of sepsis, lung injury and other inflammatory diseases^27^,^28^,^29^,^30^. Recent studies have investigated the role for TREM-1 in viral immune response. Activation of TREM-1 has been reported with Marburg, Ebola, Hepatitis B, Dengue and influenza viruses^31^,^32^,^33^. A recent study showed that gp41 protein of HIV upregulates TREM-1 in peripheral blood monocytes^34^. However, it is not established how TREM-1 activation contributes to HIV viral immune response. In a recent study we have shown that induction of TREM-1 prolongs survival of macrophages in response to lipoolysaccharide through Egr2-Bcl-2 signaling^35^. Therefore we hypothesized that activation of TREM-1 may be a mechanism by which macrophages harboring HIV viral proteins or virions become resistant to apotosis thus enhancing their persistence.

In this study we investigated the effects of HIV infection on TREM-1. Our data show that TREM-1 is induced in human monocyte/marophages infected with HIV. In parallel, the HIV-related proteins Tat and gp120 induce TREM-1 in macrophages in an NF-κB p65-dependent manner. Finally, activation of TREM-1 functionally prolongs macrophage survival, whereas silencing TREM-1 induces apoptosis in macrophages exposed to HIV-related proteins, and this is mediated through activation of caspase 3 and reduction in Bcl-2. In summary, these data suggest a novel role for TREM-1 in HIV latency within macrophages and may bear important therapeutic implications for the reduction or possibly even the eradication of persistent HIV reservoirs.

## Results

### TREM-1 expression in HIV-1-infected human monocyte-derived macrophages

**Figure 1:**
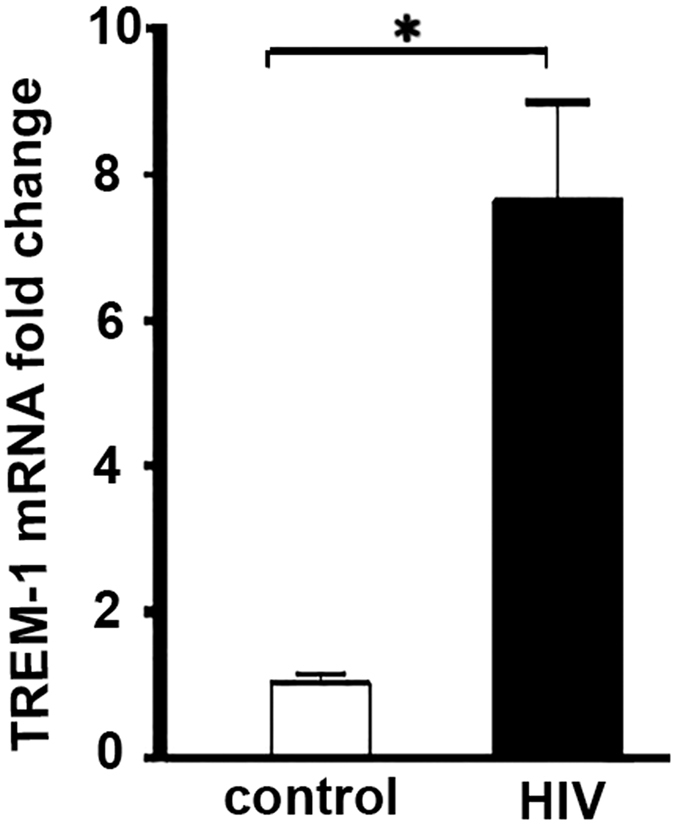
TREM-1 expression in HIV-1- infected human monocyte-derived-macrophages (MDMs). Human MDMs were infected with HIV-1 for 8 days. Total RNA was extracted for mRNA analysis. There was a 7.6-fold increase of TREM-1 expression in MDMs infected with HIV-1 compared to uninfected cells (*p < 0.05, n = 6–7).

Human monocytes were isolated from healthy volunteers as described previously^25^, and cultured in growth media for seven days and matured to macrophages. Mature macrophages were infected with HIV-1 viral particles at 2 × 10^6^ tissue culture infective dose (TCID) of 50/cell for four hours. TREM-1mRNA expression level was detected in infected and uninfected control cells. As shown in Fig. 1, macrophages infected with HIV-1 viral particles showed a significant induction in TREM-1 gene expression level compared to uninfected control cells. Total RNA was extracted from mature macrophages and expression of TREM-1 was determined by qRT-PCR. These data show that HIV-1 viral infection induces expression of TREM-1 in monocyte-derived human macrophages.

### Recombinant Tat and gp120 proteins induce TREM-1 expression in macrophages

**Figure 2:**
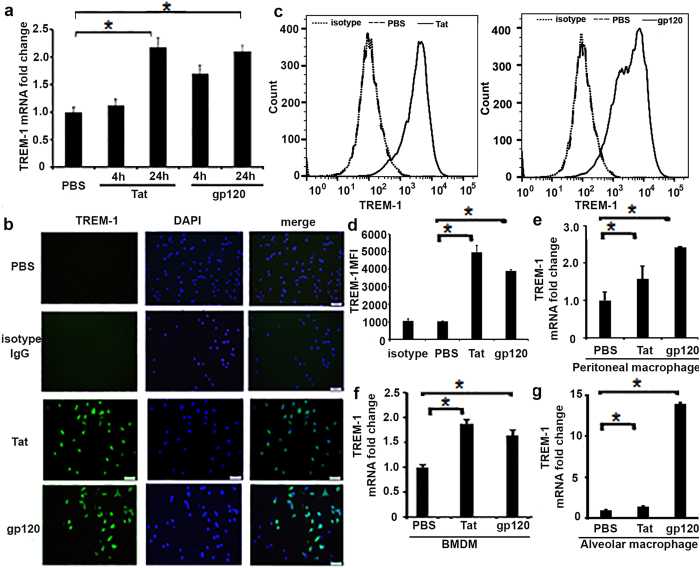
TREM-1 expression in macrophages treated with recombinant HIV Tat and gp120. RAW264.7 cells were treated with recombinant Tat (100 ng/ml) and gp120 (100 ng/ml) for 4 h and 24 h, respectively. (**a**) qPCR demonstrating an increased expression of TREM-1 at 24 hours in cells treated with Tat and gp120; (**b**) representative images of immunofluorescence staining demonstrating TREM-1 expression in RAW264.7 cells treated with recombinant Tat and gp120 (100 ng/ml) for 24 h. Fixed cells were stained with anti-TREM-1/rabbit anti- goat FITC Abs (green) and nucleus was counterstained with DAPI (blue). Original magnification, X40, scale bars, 50 μM. (**c**) representative histogram images showed increased expression of TREM-1 protein at 24 hours; (**d**) Mean fluorescence intensity (MFI) in HIV Tat and gp120-treated macrophages. Data are shown as means ± SE, *P < 0.05 as significant difference. (**e**) Peritoneal macrophages, (**f**) BMDM and (**g**) alveolar macrophages from wild type C57BL/6, were treated with HIV Tat and gp120 (100 g/ml) for 24 h, respectively. TREM-1 expression was analyzed by Q-PCR. Statistical significance was assessed by two-tailed paired Student’s test. Data are shown as means ± SE, *P < 0.05 as significant difference.

Next we wanted to determine if HIV-related proteins induce expression of TREM-1 in macrophages *in vitro*. RAW264.7 cells were treated with recombinant Tat or gp120 (100 ng/ml) for 4 and 24 hours, respectively. Cells were harvested and total RNA was extracted. Both Tat and gp120 individually induced gene expression of TREM-1 at 24 hours when compared to control cells treated with vehicle alone (Fig. 2a). We also determined the protein expression of TREM-1 in response to HIV viral protiens in macrophages. RAW264.7 cells were treated with Tat or gp120 (100 ng/ml) for 24 hours and then fixed and stained with anti-TREM-1/rabbit anti-goat FITC antibodies, and the nuclei were counterstained with DAPI. Cells treated with Tat and gp120 showed a signifiacntly increased expression of TREM-1 protein compared to control cells treated with vehicle (Fig. 2b). Expression of TREM-1 protein was determined at 24 hours in cells treated with Tat or gp120 using flow cytometry, as shown in Fig. 2c, and mean fluorecence intensity (MFI) assay also showed that expression of TREM-1 protein increased at 24 hours in cells that were treated with Tat and gp120, respectively, compared to cells that were treated with PBS (Fig. 2d). Further, we validated the expression of TREM-1 protein by immunoflourescent staining. Similar experiments were performed in peritoneal macrophages, BMDM and alveolar macrophages from wild type C57BL/6 mice. Macrophages that were treated with recombinant Tat and gp120 showed an increased expression of TREM-1 at 24 hours in all the macrophages subtypes (Fig. 2e–g). Together these data show that HIV-related proteins induce expression of TREM-1 in macrophages in a time-dependent manner.

### TREM-1 expression in macrophages induced by Tat or gp120 is NF-κB p65 dependent

**Figure 3:**
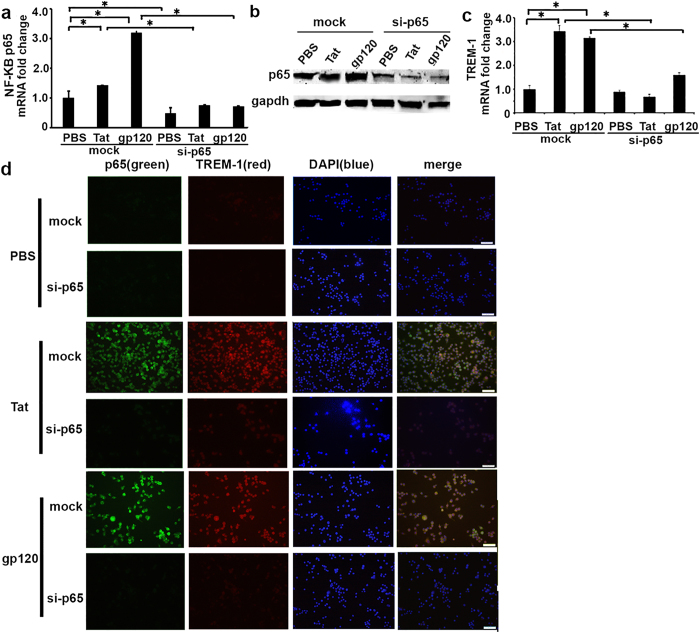
TREM-1 expression in macrophages induced by HIV Tat and gp120 is NF-κB p65 dependent. RAW264.7 cells were transfected with si-NF-κB p65 or negative control siRNA (total 100 pmol of siRNA for each transfection). 24 hours later, transfected cells were treated with recombinant Tat and gp120 for 24 hours following which cells were harvested and total RNA was extracted. (**a**) Real-time PCR and (**b**) western blot assay showed NF-κB p65 expression was significantly reduced in cells with si-NF-κB p65, compared to cells that were treated with control siRNA. Statistical comparisons were performed, numbers are mean ± SE, *P < 0.05 as significant difference; (**c**) Detection of TREM-1 mRNA expression in NF-κB p65 silenced- RAW264.7 cells treated with HIV Tat and gp120. (**d**) Representative images of RAW264.7 cells stained for NF-κB p65 and TREM-1. Cells were transfected with si-NF-κB p65 or control siRNA. Expression of TREM-1 protein was significantly attenuated in cells treated with si-p65 compared to control siRNA. Original magnification 40X, scale bars: 50 μm.

Next we wanted to investigate the mechanism by which HIV-related proteins induce expression of TREM-1 in macrophages. We have previously shown that expression of TREM-1 is transcriptionally regulated by NF-κB and PU.1 in macrophages in response to LPS^36^,^37^. In particular, NF-κB p65 binds to the TREM-1 promoter and induces expression of TREM-1 in macrophages treated with LPS, whereas PU.1 inhibits its expression^36^,^38^. In order to investigate whether NF-κB p65 regulates expression of TREM-1 in response to these HIV-related proteins, we transfected RAW264.7 cells with siRNA against p65 or control siRNA for 24 hours. Cells were then treated with recombinant Tat or gp120 (100 ng/ml) for 24 hours. Total RNA was extracted and qRT-PCR and protein expression by western blotting was perfromed to determine the expression of p65. Upon treatment with Tat or gp120, expression of p65 NF-kB gene and protein was induced in cells treated with Tat or gp120 (Fig. 3a and b). Knockdown of p65 was confrimed in cells with si-p65. Next we performed experiments to determine the expressio of TREM-1 message and protein in cells with si-p65. TREM-1 was induced in cells that were transfected with mock siRNA, however, expression of TREM-1 was significantly attenuated in cells that were transfected with si-p65 (Fig. 3c).

Similar experiments with sip65 were performed to determine expression of TREM-1 protein by immunoflourescence staining after 24 hours of treatment with Tat or gp120 (100 ng/ml). As shown in Fig. 3d, TREM-1 protein expression was induced in macrophages that were transfected with control siRNA and treated with Tat or gp120. However, expression of TREM-1 protein was inhibited in cells that were transfected with si-p65. Together, these data suggest that expression of TREM-1 in response to these HIV-related proteins is regulated by p65 in macrophages.

### Tat and gp120 prolong macrophage survival through TREM-1 induction in macrophages

**Figure 4:**
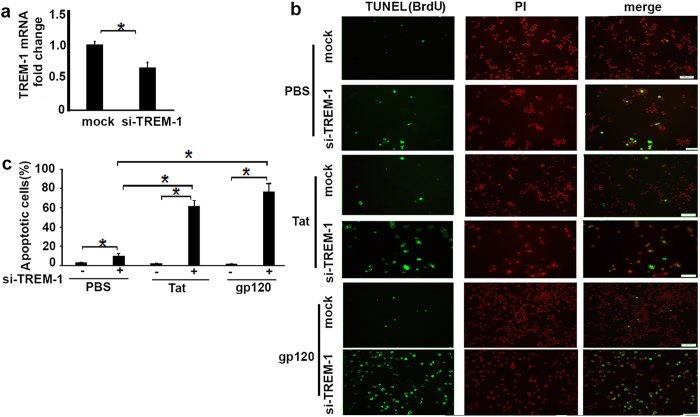
Macrophage apoptosis in TREM-1 knockdown cells treated with recombinant HIV Tat and gp120. RAW264.7 cells were transfected with si-TREM-1 or control siRNA for 24 hours following which they were treated with recombinant HIV Tat and gp120 (100 ng/ml).(**a**) TREM-1 expression was significantly reduced in cells with si-TREM-1, compared to cells that were treated with control siRNA. (**b**) cell apoptosis determined by TUNEL staining (green) and PI (red) scale bars, 50 μM, (**c**) Percentage of TUNEL-positive cells per DAPI-positive cells were measured from 200 cells for each preparation from three independent experiments, *p < 0.05 considered significant. Statistical comparisons were performed, numbers are mean ± SE, *P < 0.05 as significant difference.

We have previously shown that TREM-1activation inhibits macrophage apoptosis and promotes cell survival signaling in response to LPS^35^. Since HIV-infected macrophages are resistant to apoptosis^39^,^40^, and our data show that HIV induces expression of TREM-1, we hypothesized that TREM-1 signaling may contribute to the prolonged survival of HIV-infected macrophages. We first assessed Tat- and gp120-induced apoptosis in TREM-1 knockdown cells using siRNA against TREM-1. We reasoned that if TREM-1 is involved in promoting cell survival, then elimination of TREM-1 should increase cell death. RAW264.7 cells were transfected with control siRNA or TREM-1 siRNA using nucleofaction for 24 hours. Knockdown of TREM-1 was confirmed in cells with siTREM-1 (Fig. 4a). Transfected cells were treated with Tat or gp120 (100 ng/ml) for 24 hours, and stained with Alexa Fluor 488 dye-labeled anti-BrdU antibody (green) and PI (red). Percentage of TUNEL positive cells per DAPI-positive cells were measured within 200 cells for each area. TREM-1 knockdown cells showed an increase in apoptosis, compared to cells that were transfected with control siRNA (Fig. 4b and c). These data suggest that induction of TREM-1 by viral proteins may confer anti-apoptotic attributes to macrophages and thereby allowing the virus to establish an intracellular reservoir.

### Silencing TREM-1 increases caspase-3 activity and inhibits Bcl-2 expression

A central step in the execution of apoptosis is the activation of caspases, a family of cysteine proteases that are ubiquitously expressed as inactive precursors with little or no protease activity^41^,^42^. The caspase family is subdivided into initiator and effector caspases. Effector caspases execute apoptosis after they are proteolytically processed by initiator caspases. We have previously shown that in response to LPS, TREM-1 knockdown cells show an increase in executionary caspase-3 with cleavage of PARP in response to LPS^35^. Further, our data indicate that TREM-1 knockdown in macrophages renders them susceptible to apoptosis when exposed to HIV-related proteins. Therefore, we next determined the effects of TREM-1 signaling on caspases in macrophages treated with Tat and gp120.

**Figure 5:**
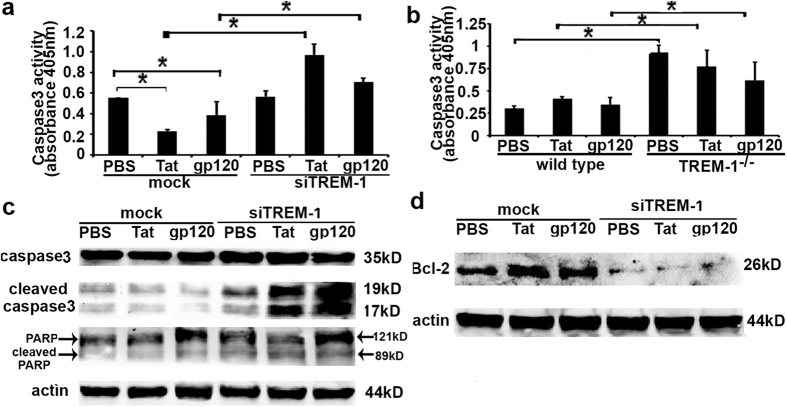
Capsase-3 activity and Bcl2 expression in TREM-1- silenced macrophages treated with recombinant Tat and gp120 protein. RAW264.7 cells were transfected with siRNA against TREM-1 or control siRNA for 24 hours and then treated with recombinant HIV Tat and gp120 (100 ng/ml) respectively. (**a**) Caspase3 activity was measured in RAW264.7 macrophages. (**b**) Caspase3 activity was also measured in BMDM from wild type C57BL/6 and TREM-1/3 deficient mice, *p < 0.05. Caspase3, cleaved caspase3, PARP and cleaved PARP expression, shown in (**c**), and Bcl-2 expression, shown in (**d**), were analyzed by western blot, and actin was used as internal control.

To determine the effects of TREM-1 on activity of caspase-3, we transfected RAW264.7 cells with TREM-1 siRNA or control siRNA for 24 hours prior to treating them with recombinant Tat or gp120. Caspase 3 activity was determined by Caspase-3/CPP32 colorimetric assay. Activity assay showed that Tat and gp120 inhibited caspase 3 activity in cells transfected with control siRNA, whereas cells that were transfected with si-TREM-1 showed an increase in caspase-3 activity (Fig. 5a), which contributes to the increase in apoptosis of TREM-1 knockdown macrophages. To further confirm the role of TREM-1, we treated BMDM from TREM-1/3 deficient mice^29^ with Tat or gp120 (100 ng/ml) for 24 hours. In agreement with our results from TREM-1 knockdown cells, we found an increase in caspase-3 activity from TREM-1 knockout BMDM that were treated with Tat or gp120 (Fig. 5b). To further determine the effects of TREM-1 on executionary caspases, TREM-1 knockdown cells and control cells were treated with Tat or gp120 (100 ng/ml). Caspase-3, cleaved caspase-3, PARP, and cleaved PARP expression were determined by Western blotting. We found that TREM-1 knockdown cells showed an increased expression of cleaved caspase-3 and PARP (Fig. 5c) suggesting that the presence of TREM-1 is necessary for the functional activation of caspase-3. We also determined the effects of TREM-1 knockdown on the key anti-apoptotic protein Bcl-2. Cells treated with siTREM-1 or TREM- knockout macrophages showed a significant decrease in the expression of Bcl-2 in response to Tat and gp120 protein (Fig. 5d). Together these data show that Tat and gp120 inhibit apoptosis of macrophage through TREM-1 signaling by increasing caspase 3 activity and inhibiting Bcl-2.

## Discussion

This study provides novel evidence that HIV infection induces the expression of TREM-1 in human macrophages. We also determined that the HIV-related proteins Tat and gp120, which circulate in individuals living with HIV and diffuse into tissue compartments including the airways, likewise induce TREM-1 even in the absence of HIV infection *per se*, and this is mediated through an NF-κB p65-dependent mechanism. Moreover, the induction of TREM-1 by these HIV-related proteins inhibits the activation of caspase 3 suppressing macrophage apoptosis. These results are provocative, as they suggest that in a microenvironment such as the alveolar space, these HIV-related proteins can induce an ‘anti-apoptotic’ phenotype in macrophages that would render them more hospitable hosts to subsequent infection by HIV and thereby maintain and/or propagate a reservoir that is resistant to eradication by current anti-retroviral drugs. Therefore, therapeutic strategies targeting this aberrant induction of TREM-1 might facilitate the clearance or at least reduction of such HIV reservoirs.

TREM-1 is a myeloid specific protein that plays a key role as an amplifier of inflammation both in the setting of infection and in non-infectious inflammation^20^,^21^. This study demonstrates that TREM-1 is induced in human macrophages infected with HIV. However, the HIV-related proteins Tat and gp120 can independently induce expression of TREM-1 in macrophages in an NF-κB p65-dependent manner. Functionally, activation of TREM-1 prolongs survival of macrophages rendering them resistant to apoptosis. Importantly, RNA silencing in our studies confirmed that TREM-1 knockdown in macrophages increased apoptosis in response to these HIV-related proteins, suggesting that activation of TREM-1 may be a mechanism by which HIV prolongs macrophage survival and promotes persistence of a retroviral reservoir.

Although antiretroviral therapy suppresses viral replication in lymphoid tissue and has achieved considerable success at combating HIV viral infection, it is becoming evident that clinically latent reservoirs of HIV persist in individuals on antiretroviral therapy^4^,^5^,^8^,^43^. Retroviral persistence has been reported in brain cells including microglia, astrocytes, mononuclear cells, dendritic cells, CD4 lymphocytes, perivascular and tissue macrophages^5^,^44^,^45^. In general, HIV principally targets CD4^+^ T cells and cells of monocyte/macrophage lineage. Studies have also shown that in contrast to lymphocytes, macrophages exposed to HIV are resistant to apoptosis^17^. Notably, cells of monocyte/macrophage lineage are more resistant to the cytopathic effect of HIV and can harbor virus for prolonged periods of time^45^. Besides hosting a significant virus reservoir, chronically infected macrophages can contribute to disease pathogenesis through chronic aberrant release of a variety of host and viral cytoactive factors^46^,^47^. However, the molecular mechanisms of viral persistence in macrophages are not fully characterized.

HIV stimulates complex responses within macrophages through alteration of expression or modification of cellular genes and proteins^43^,^47^. While the virus modulates multiple signaling pathways over the course of infection, HIV has a selective impact on macrophage immune response. In HIV-infected individuals, macrophages manifest defective activity in their interactions with a wide variety of opportunistic pathogens, including bacteria, fungi and protozoa^48^. Macrophages infected with HIV are unable to respond efficiently to phagocytic triggers to clear pathogens^49^. Further, infection of macrophages with HIV not only modifies the level of background activation of monocytes and macrophages, but also the type of activation.

Members of the TREM signaling proteins are cell surface activating receptors that signal through adaptor protein DNAX-activating protein (DAP-12). TREM-1 is a myeloid specific receptor which plays a key role in amplification of immune inflammation^19^,^20^,^21^. Most studies have focused on non-viral infections, but recent studies have shown that TREM-1 signaling may also play a critical role in viral innate adaptive response^31^. By employing viral PAMPS, studies have shown that TREM-1 is activated in response to TLR9 ligand CpG DNA and poly (I:C) with increased production of TNF-αby human peripheral blood monocytes^21^.

Viruses that have shown to increase the expression of TREM-1 mRNA and soluble TREM-1 include filoviruses (Marburg and Ebola), flaviviruses (human DENV, WNV). However it is not established if activation of TREM-1 is pathogenic or protective in viral infections^31^. Weber *et al*. recently showed that TREM-1 knockout mice are protected from severe influenza disease although the viral clearance per se was unaltered in the knockout animals. Their study implies that limiting inflammation by blocking TREM-1 in the setting of acute viral infection may contribute to its protective effect^50^. Denner *et al*. investigated the effects of recombinant gp41 and a synthetic peptide corresponding to a highly conserved domain in gp41, the immunosuppressive (isu) domain on inflammatory response on peripheral blood monocytes. They showed that incubation of peripheral blood monocytes with isu peptide increased the expression of several pro-inflammatory genes including cytokines such as IL-1β, MMP-1 and TREM-1 suggesting a role for these proteins in immunopathogenesis of HIV^34^.

Our study is the first to show that TREM-1 is induced in response to the HIV-related proteins Tat and gp120. Macrophages treated with recombinant Tat and gp120 showed increased TREM-1 mRNA and protein. Induction of TREM-1 by HIV viral proteins was abrogated in macrophages treated with siRNA against p65 implying that the induction of TREM-1 is p65-dependent. By employing a gene silencing approach, we show that TREM-1 knockout cells demonstrate enhanced apoptosis in response to HIV viral proteins with a decrease in Bcl-2 and enhanced caspase 3 activity. Apoptosis is a self-destructive cellular process that has pivotal roles in tissue development and immune regulation^41^,^42^. In particular apoptosis of pro-inflammatory cells enhances resolution of inflammation and clearance of pathogens that seek intracellular respite in immune cells. Previously we have shown that TREM-1 inhibits apoptosis of macrophages in response to *lipopolysaccharide* by inducing Bcl-2 in Egr2 dependent manner^35^. Further we have also shown that activation of TREM-1 promotes mitochondrial integrity through induction of mitofusins^35^. Whether these pathways contribute to prolonged macrophage survival in response to HIV needs further investigation.

In summary, data from our study provide new insights for TREM-1 activation in HIV-infected macrophages and suggest that it may play a role viral latency in macrophages. TREM-1 knockout macrophages exhibited an increase in apoptosis in response to HIV viral proteins. Inhibition of macrophage apoptosis may contribute to viral latency and persistence in HIV infected individuals. Thus these data suggest that TREM-1 activation on macrophages infected with HIV may be a mechanism by which HIV persist in macrophages. Moreover, these studies provide novel insights into the role of TREM-1 activation in HIV infection and suggest that TREM-1 activation may contribute to dysregulated macrophage immune responses. Collectively, our study highlights a novel therapeutic role for blockade of TREM-1 to enhance clearance of otherwise resistant HIV reservoirs in macrophages.

## Materials and Methods

### Chemicals and reagents

Recombinant HIV IIIB Oligomeric Glycoprotein gp120 (Baculovirus)(product#1061), and Recombinant tat HIV-1IIIB (product#1002) were obtained from ImmunoDX (USA). Primary antibody against mouse TREM-1(MAB1187) was purchased from R&D systems (USA). Antibodies against caspase3, cleaved caspase3, PARP, cleaved PARP and Bcl-2 were obtained from Cell Signaling Technology (USA), and all other antibodies were purchased from Santa Cruz Biotechnology (USA).

### Animals

Wild type C57BL/6 mice were purchased from Jackson Laboratories (USA). TREM-1/3-deficient mice were kindly provided by Dr. Marco Colonna (Washington University at St. Louis) (29). All procedures were approved by the Institutional Animal Care and Use Committee at Emory University. All methods were performed according to the approved guideline consistent with American Veterinary Medical Association.

### Cell culture and treatment

Human monocyte-derived-macrophages (MDMs) were isolated from peripheral blood from healthy human volunteer donor after obtaining informed consent approved by the Institutional Review Board and in accordance with the Ethical Human Guidelines for research. MDMs were isolated from the buffy coat by Ficoll centrifugation as described previously^25^,^51^. In brief, after centrifugation cells were seed at density of 1–2 × 10^5^/well in 24-well plate coated with poly-D-Lysine (Sigma-Aldrich, St. Louis, MO) in serum-free RPMI-1640 at 37 °C, 5% CO_2_ for 30 min and then non-adherent cells were removed prior to culture in RPMI-1640 with 10% FBS, 1% non-essential amino acid, 1% Sodium pyruvate (growth medium). After seven days, cells were infected with or without HIV-1 viral particles at 1–2 × 10^6^ TCID50/cell for four hours and then replaced with fresh growth medium and continue to culture for 8 days. The protocol was approved by Emory University Institutional Review Board. Mouse bone marrow-derived macrophages (BMDM) from C57BL/6 J and TREM-1/3-deficient mice were prepared as described previously^29^,^38^. Mouse alveolar macrophages and peritoneal macrophages were harvested from C57BL/6 mice, as described previously^52^. RAW264.7 cell line was purchased from ATCC (USA) and cultured in DMEM supplemented with 10% FBS at 37 °C, 5%CO_2_. For the transfection assays, cells were transfected with siRNA against TREM-1 (100nmol/L, Life technologies, USA), siRNA against NF-kB p65 and *mir*Vana^™^ Negative Control (100 nmol/L, Life technologies, USA) through a specific LONZA transfection reagent (LONZA, USA) according to the manufacturer’s instructions, as described previously^35^.

### Flow Cytometry

Cells were harvested and incubated with FcR blocking reagent (Miltenyi Biotec, USA), then incubated with rat anti-mouse TREM-1 phycoerythrin conjugated monoclonal antibody (R&D, Catalog # FAB1187P) or rat IgG2a phycoerythrin Isotype Control (R&D, Catalog # IC006P), 30 minutes at 4 °C. All samples were acquired on Becton Dickinson FACSAria™ II (Beckman, USA) and analyzed with FlowJo (Tree Star, USA).

### Quantitative Real-Time RT-PCR

RNA from sorted cell populations was isolated with Trizol reagent (Invitrogen, USA), and reverse-transcribed to cDNA using the First Strand cDNA Synthesis Kit (MBI Fermentas, USA) according to manufacturer’s instructions. Real-time RT-PCR was performed using Taqman PCR mix and primers (Life technologies, USA). Reactions were run using the 7500 PCR System (Life technologies, USA). The results were displayed as relative expression values normalized to actin.

### TUNEL assay

*In vitro* apoptosis assay was measured by Terminal Deoxynucleotide Transferase dUTP Nick End Labeling (TUNEL) method, using APO-BrdU™ TUNEL Assay Kit (Molecular probes, USA) through labeling 3′-end of fragmented DNA of the apoptotic cells. TUNEL assay was performed according to the manufacturer’s instructions. The transfection was performed as described previously^35^. After treatment, cells were harvested and fixed with 1% paraformaldehyde and cold 70% ethanol, then added DNA-labeling solution containing BrdUTP, and incubated with Alexa Fluor 488 dye-labeled anti-BrdU antibody, finally counterstained with propidium iodide. All slides were observed under a fluorescent microscopy. The stained cells were examined under Olympus FV1000 confocal microscope (Japan). For quantitative results, the TUNEL positive cells were counting in three random area images for each sample by Image J program (NIH, USA), 200 cells were successively counted for each field by an observer who did not identify the slides. The ratio of TUNEL-positive cell number to the total cell number is shown.

### Caspase-3 activity assay

Caspase-3/CPP32 Colorimetric assay kit was used to determine caspases activity according to the manufacturer’s instructions (BioVision, USA). Cells were treated and collected with cell lysis buffer. Samples were read at 405 nm at a microtiter plate reader.

### Immunofluorescence staining

Cells were fixed in 1% (vol/vol) paraformaldehyde for 15 min at 4 °C, washed in PBS, and then blocked in PBS with 0.2% Triton X-100 containing 10% (vol/vol) normal donkey serum for 1 h at room temperature. Primary antibodies were incubated overnight at 4 °C, and followed by secondary antibody incubation with FITC goat anti-rabbit IgG (Santa Cruz, USA) and Texas Red rabbit anti-goat IgG (Santa Cruz, USA) for 1 h, room temperature. Slides were mounted in Vectashield containing DAPI (Vector Laboratories, H-1200, USA). All slides were visualized by using Olympus FV1000 confocal microscope (Japan).

### Western blotting

Samples were prepared in RIPA buffer containing proteinase and phosphatase inhibitors according to instructions (Sigma Aldrich, USA). Western blotting was performed as described previously^53^, appropriate primary antibodies and horseradish peroxidase-conjugated secondary antibodies were used prior to visualization via chemiluminescence (Amersham Biosciences, USA).

### Statistical analyses

GraphPad Prism version 6.0 (GraphPad) was used for statistical analysis. Student’s t-test was used to compare the means of data from two experimental groups while significant differences (p < 0.05), and multiple groups were confirmed by one-way ANOVA and Tukey’s post hoc multiple comparisons testing. Results are expressed as means ± SE, P < 0.05 was considered significant.

## Additional Information

**How to cite this article**: Yuan, Z. *et al*. HIV-related proteins prolong macrophage survival through induction of Triggering receptor expressed on myeloid cells-1. *Sci. Rep.* **7**, 42028; doi: 10.1038/srep42028 (2017).

**Publisher's note:** Springer Nature remains neutral with regard to jurisdictional claims in published maps and institutional affiliations.
